# Effect of Lycosome-Formulated Phosphatidylcholine on Parameters of Biological Oxidation after Single Intake of Moderate Amount of Alcohol

**DOI:** 10.1155/2018/5840451

**Published:** 2018-07-30

**Authors:** Ivan M. Petyaev, Marina P. Chernyshova, Dmitry V. Pristensky, Natalia E. Chalyk, Victor A. Klochkov, Nigel H. Kyle, Yuriy K. Bashmakov

**Affiliations:** ^1^Lycotec Ltd., Granta Park Campus, Cambridge CB21 6GP, UK; ^2^Institute of Cardiology, 12 Chernyshevskogo Str., 410028 Saratov, Russia

## Abstract

Ingestion of a single dose of alcohol, ranging from the intake of a moderate amount alcohol to binge drinking, is the most frequent form of alcohol consumption with poorly understood medical consequences and obscure prophylactics. The study was aimed to determine whether lycosome formulated phosphatidylcholine (PC-Lyc) containing two highly bioavailable antioxidants (PC and lycopene) ingested shortly before the alcohol-containing beverage may alleviate the biochemical markers of liver damage and parameters of biological oxidation associated with the intake of a moderate amount of alcohol. Healthy middle-aged volunteers were requested to consume a moderate amount of alcohol – 0.5 ml/kg or 1.0 ml/kg shortly after ingestion of a capsule containing 450 mg of regular phosphatidylcholine (PC, n=10), PC-Lyc (n=10), or placebo pill (PP, n=10). Serum levels of ethanol (EtOH), acetaldehyde (AA), liver-specific enzymes, total antioxidant capacity of serum (TAC), oxidized LDL (LDL-Px), and malonic dialdehyde (MDA) were measured at 1, 2.5, and 5 hours after dosing with alcohol. Ingestion of PC regardless of the formulation used had no effect on serum EtOH concentration dynamics. However, volunteers supplemented with PC-Lyc showed a better clearance of AA in serum as compared to other groups. There was a reduction in serum TAC values by 18.5% and 16.1% in both placebo groups ingesting 0.5 and 1.0 ml/kg of alcohol, respectively, at the end of observational period. This decline was preventable by supplementation of volunteers with PC and especially with PC-Lyc. Moreover, PC-Lyc promoted a reduction of serum MDA and reversed an increase in serum LDL-Px. In addition, ingestion of alcohol at 1.0 ml/kg dose caused a transient increase in serum alanine-aminotransferase activity which was abolished by both formulations of PC. Therefore, combinatory lycosomal formulation of PC and lycopene may prevent some metabolic abnormalities associated with single intake of moderate amount of alcohol. This trial is registered with ACTRN12617001335381.

## 1. Introduction

Alcohol consumption may be associated with multiple detrimental health effects including the alcoholic liver disease (ALD). Excessive alcohol intake causes 4.5% of common diseases and accounts for 4% of mortality in the general world population, while in Eastern European countries it is a major cause of death among men aged 15-59 years [1–3]. The liver is a unique organ controlling metabolic response to alcohol. Hepatocytes are the major cell type supporting ethanol metabolism and paradoxically can become a major cellular target for ethanol toxicity. In general terms, the liver is the first gate-keeper for all nutrients, electrolytes, xenobiotics, and toxic substances absorbed in the gastrointestinal tract. Following alcohol intake the portal blood contains 2-3 times more ethanol than blood from the systemic circulation [4]. Moreover, the liver is the major organ of ethanol bioconversion pathways known to produce various toxic ethanol intermediates including acetaldehyde [5].

Alcohol is considered to be a potential precipitating etiogenic factor causing acute, chronic, or acute-on-chronic liver failure [6]. Alcoholic liver disease (ALD) is associated with high morbidity as well as mortality fluctuating from 30 to 50% worldwide with the severity ranging from asymptomatic derangement of liver biochemistry parameters to liver failure or death [7]. Rapidly progressing jaundice and coagulopathy accompanied by encephalopathy are the most common symptoms of acute alcoholic and acute-on-chronic liver failure [7, 8]. Chronic forms of ALD are represented by a spectrum of pathological conditions ranging from steatosis (fatty liver) to steatohepatitis and in the most severe cases by liver fibrosis, cirrhosis, and hepatocellular carcinoma [9, 10].

The pathogenesis of ALD is still poorly understood [8]. The direct effects of ethanol and especially its main toxic intermediate, acetaldehyde, on liver cells were under the scope of the majority of investigators for decades. However, recent progress in molecular science has unveiled the pivotal role of an ethanol-induced inflammatory response initiated by an increased gut permeability and endotoxin portal influx which leads to the subsequent activation of macrophages and Kupffer cells resulting altogether in increased production of cytokines [11, 12]. Therefore, besides abstinence from alcohol, anti-inflammatory interventions become the first choice option in management of ALD [11, 13]. A deeper insight has also been gained recently into the potential role of reactive oxygen species (ROS) and antioxidants in the etiology, pathogenesis, and outcomes of ALD [14, 15]. This fuels the recent interest of many researchers in antioxidant use for the treatment of ALD [15, 16]. Although ingestion of a single dose of alcohol, ranging from the intake of a moderate amount alcohol to binge drinking, is the most frequent form of alcohol consumption, its medical consequences are studied and understood much less than the pathogenesis and treatment of ALD. At present, no medical or pharmacological recommendations are clearly defined for occasional alcohol intake.

In the present paper we report that ingestion of a novel lycosome-phospholipid nutraceutical formulation containing lycopene, an antioxidant, and phosphatidylcholine (PC), a membrane-stabilizing phospholipid, ameliorates some abnormalities of biological oxidation in healthy volunteers following single intake of a moderate amount of alcohol and can be used for the prevention of alcohol-induced complications following occasional alcohol intake.

## 2. Materials and Methods

The study was initiated and conducted by Lycotec Ltd. (Cambridge, UK) at its facilities in Cambridge, UK, and at the Institute of Cardiology, the Ministry of Health of the Russian Federation (Saratov, Russian Federation). The study was conducted under a protocol approved by the local Ethics Committee and registered (ACTRN12617001335381). The study was designed as a single, interventional, randomized, crossover, placebo-controlled study to determine whether lycosome-formulated phosphatidylcholine (PC-Lyc) ingested shortly before the alcohol-containing beverage may alleviate the biochemical markers of liver damage (plasma activity of liver-specific enzymes) and parameters of biological oxidation (total antioxidant capacity of serum, oxidized LDL, and Inflammatory Oxidative Damage) associated with the intake of a moderate amount of alcohol. Therefore the end points of the study included the values for ethanol (EtOH) and acetaldehyde (AA) concentration in serum and parameters of biological oxidation and serum liver-specific enzymes during the recovery phase (1, 2.5, and 5 hours) after acute intake of alcohol following ingestion of lycosome-formulated phosphatidylcholine (PC-Lyc) or PC alone. All volunteers were fully informed about the purpose of the study and its specific aims as well as its outcomes and signed a written consent form regarding their participation. All volunteers went through physical and laboratory examinations and were asked about their medical history and socioeconomic situation. All medical evaluations were performed in a window of time from 0-30 days before study initiation. Evaluations included the vital signs, determination of body mass index, anthropometry, blood pressure measurement, and electrocardiography. Among the laboratory tests were determination of fasting serum glucose and lipids (total cholesterol, LDL cholesterol, HDL, and total triglyceride levels), determination of ALT and AST levels, total bilirubin, and hs-CRP. All volunteers were also the subject of a standard hematological investigation. The volunteers were requested to abstain from consumption of alcohol and any nutritional supplements for 2 weeks prior to the study and were subjected to alcohol intake strictly as mandated by the study protocol. Only volunteers with undetectable serum levels of ethanol and acetaldehyde at the zero time point of the study were allowed to participate in the trial.

The volunteers were randomized according to their age, gender, body weight, and BMI using a simple randomization method and block randomization.

### 2.1. Subjects and Inclusion/Exclusion Criteria

Subjects were healthy Caucasian males and females with no history of extensive alcohol use or liver disease aged from 40 to 60 years and body weight from 60 to 90 kg and healthy body mass index (BMI, 19-24).

#### 2.1.1. Major Inclusion Criteria 

They were as follows: Caucasian male or female subjects 30-60 years old, absence of concomitant intake of antihypertensive, lipid-lowering or any other cardiovascular drugs, vitamin supplements or any specific dietary interventions, and no anamnestic indication of heavy alcohol use.

#### 2.1.2. Major Exclusion Criteria 

They were as follows: inability to comply with the study protocol, severe medical conditions (hepatitis, pancreatitis, uncontrolled diabetes, cancer, recent cardiovascular events, tuberculosis, etc.), history of alcohol abuse/alcoholism, and tomato and egg allergies.

### 2.2. Study Protocol

#### 2.2.1. Wash-Out Period

The volunteers were asked to refrain from consumption of alcohol and tomato based products for 14 days before beginning the study.

#### 2.2.2. Alcohol Intake

The interventional procedure was conducted strictly according to the conditions described in the approved protocol. The volunteers were requested to abstain from any food intake and arrive at the study site for a standardized breakfast in the fasting state at 9.00 am. Upon arrival at the study site all volunteers were given a standardized breakfast which included 2 scrambled eggs (total mass 122 gr, 186 kcal, 1.2 g of carbohydrates, 12.6 g of protein, and 14 gr of fat) with 2 slices of bread (total mass 50 gr, 134 kcal, 24.8 g of carbohydrates, 4 gr of protein, and 0.18 gr of fat). Breakfast was served hot and prepared by trained food workers prior to arrival of volunteers at the study site and carefully weighed. Participants were requested to ingest all breakfast food. Study products were given immediately after the standardized breakfast.

Dosing with alcohol took place 1 hour after the standardized breakfast and ingestion of investigational product. The volunteers were offered 240 ml of alcohol-containing beverage containing lemonade and alcohol. To make lemonade 1000 ml of Nestle drinking water was mixed with 2 crushed lemons. The mixture was kept overnight at 4°C, filtered, and used for the study as a cold beverage. Alcohol was served at doses of 0.5 gr/kg or 1.0 gr/kg in individually labeled plastic bottles giving consideration to group assignment and adjusted to the body weight of each volunteer. For making alcohol-containing lemonade a 98% solution of alcohol made from corn (Everclear-R, Luxco, USA) was used. All bottles were labeled with the names of the participants and kept at 4°C before serving. Volunteers from all groups were asked to consume the lemonade beverages with alcohol at the same time within 15 minutes. From this time point the countdown of an observational period was designed to begin. The duration of the observational period was 5 hours.

#### 2.2.3. Observational Period

During the observational period all study participants were allowed to drink regular water only. No food intake was allowed. Throughout the observational period (prior to alcohol intake as well as at 1, 2.5, and 5 hours after dosing with alcohol) blood specimens were collected and processed for biochemical analysis. Serum was frozen at – 80°C for later analysis.

### 2.3. Study Groups

The study was conducted using 30 healthy volunteers divided into three groups containing 10 volunteers each. Individuals from the first subgroup were assigned to receive one hour prior to alcohol dosing a placebo capsule, and the subjects from the second group were assigned to receive one hour prior to alcohol dosing a capsule containing regular formulation of phosphatidylcholine (PC). Volunteers from the third subgroup were given a capsule containing lycosome formulation of PC. There were two alcohol challenges performed by oral intake of the alcohol-containing beverage. During the first challenge volunteers were given an alcohol-containing drink equivalent to 0.5 ml of ethanol/kg of body weight. After the five-hour observational period and blood sampling the volunteers were dismissed for 10 days (recovery period) and were asked to attend the study site for the second alcohol challenge performed by oral intake of alcohol-containing beverage at a dose of 1.0 ml ethanol/kg of body weight. The biochemical parameters of serum at the zero time point were taken into consideration to select retrospectively qualifying individuals. Four volunteers showed some deviations in health status and/or in serum biochemical values at the second challenge which were not related to the study specifics (respiratory infections). These individuals were replaced with qualifying volunteers from a preselected pool of volunteers. All parameters obtained during the observational period were compared to the basal control values acquired from each study participant on the same day as intervention. No “historic” controls were allowed to be used in the analysis of results following the second challenge with alcohol.

### 2.4. Study Products

#### 2.4.1. Placebo

Placebo group participants received a capsule containing 450 mg of inert, irrelevant, and nonabsorbable compound.

#### 2.4.2. Regular Formulation of Phosphatidylcholine (PC)

The participants ingested a capsule with 450 mg of phosphatidylcholine (PC, pharmaceutical grade) obtained from Lipoid GmbH, Germany.

#### 2.4.3. Lycosome Formulation of Phosphatidylcholine (PC-Lyc)

Participants were assigned to take a capsule containing 450 mg of PC as lycosome formulation containing 7 mg of lycopene (Lycotec, Cambridge, UK). The lycopene used for formulation of the PC lycosomes was in the form of tomato oleoresin from Lycored Inc. (NJ, USA) and contained 97% of all trans-isomers and 3% of all cis-isomers.

Lycosome formulation of PC is a proprietary formulation of PC (Lycotec Ltd., Cambridge, UK) with enhanced bioavailability of phospholipid in which phospholipid is protected from oxidation by a lycopene layer. Lycopene-based lycosomes [17, 18] provide a significant degree of protection to cargo molecules from stomach acidity and intestinal enzymes increasing thereby the bioavailability of PC and its intestinal absorption rate. As we have shown previously [17–24], lycosome microencapsulation enhances bioavailability and intestinal absorption rate of some amphiphilic nutraceuticals (resveratrol, whey protein peptides, and cocoa polyphenols) and pharmaceuticals (HMG-CoA reductase inhibitors).

All individual packages containing PC formulations were labeled with a numerical code and shipped to the study site. None of the volunteers were informed about the study group assignment or the dispensed product specification for the duration of the study period.

### 2.5. BMI, Pulse Rate, and Blood Pressure

Body mass index (BMI) was calculated as described elsewhere. Pulse rate and systolic and diastolic blood pressure were measured three times in the left arm of seated volunteers following 15 minutes of rest. The time between measurements was no less than 2 minutes. The mean value for each parameter was calculated. All parameters were measured in the morning between 8 and 10am.

## 3. Analytical Procedures

### 3.1. Ethanol Measurements in Serum

A gas chromatography with flame ionization detection method (GC–FID) with direct injection and a capillary column was used for ethanol (EtOH) measurements in serum [25]. One hundred microliters of serum sample was mixed with 10 *μ*L of IS (2.2 g/L 1-propanol in deionized water). The samples were diluted to 500 *μ*L with Triton X-100 solution (1,2% in deionized water). Each tube was vortex-mixed and centrifuged at 16,000 g for 5 min at 4°C. A fixed volume of supernatant (0.5 *μ*L) was injected into the GC. The GC used was a Shimadzu 2010 GC, the injection port of the chromatograph was fitted with a glass liner (5-mmi.d.) appropriate for split analysis, and the liner was replaced after 50 injections. The analyses were performed under the following chromatographic conditions: column, Supelcowax 10, 30 m × 0.32 mm i.d., and DF = 0.25 *μ*m. The temperature of the FID was 220°C, and the injector temperature was 220°C. The oven temperature was programmed to 40°C (for 1 min), followed by an increase of 5°C/min up to 70°C, followed by an increase of 20°C/min up to 200°C. The carrier gas was helium with a flow rate of 1.5 mL/min. The split ratio was 100.

### 3.2. Acetaldehyde Determination in Serum

Acetaldehyde in serum specimens was measured by a combination of high performance chromatography and solid-phase extraction as described [26]. An aliquot of the chilled serum (0.1 mL) was deproteinated with 0.3mL of 3M perchloric acid on ice, followed by the immediate addition of 0.8mL of 3M sodium acetate. Following centrifugation, the supernatant was recovered and mixed with 0.5 mL of 2mM DNPH solution (0.1M acetate buffer (pH 4)–DMSO = 16:9), and the mixture was then allowed to react for 10 minutes at room temperature. A 2ml aliquot of n-hexane was added and the tubes vortex mixed for 60 s and centrifuged at 3000g for 2 min. The n-hexane layer was then carefully transferred to another vial and evaporated to dryness. The samples were redissolved in 300 *μ*l of 50% acetonitrile and injected into a UPLC Acquity BEH C18 1,7 *μ*m VanGuard precolumn (Waters, USA) which was run at 40°C in this analysis. The quantitative determinations were performed isocratically at a flow rate of 0,3 mL/min. The mobile phase consisted of acetonitrile and 0,2% trifluoroacetic acid solution in water (45:55). The ACH–DNPH peaks were detected at an absorbance of 363 nm with a diode-array detector.

### 3.3. Laboratory Parameters

Total cholesterol (TC), triglycerides (TG), HDL/LDL cholesterol, glucose, and C-reactive protein (CRP) were measured using a Biosystem A25 automated analyzer (Applied Biosystems, Grand Island, NY) using BioSys kits and calibrators.

### 3.4. Total Antioxidant Capacity (TAC)

Frozen serum specimens were analyzed within 10 days of collection using Biorex reagents according to the manufacturer's instructions (Biorex Diagnostics, Antrim, UK). Results were expressed as mmol of trolox equivalent (TE) per liter (mM TE/L).

### 3.5. Inflammatory Oxidative Damage (IOD)

Serum samples were incubated overnight in 0.05 M PBS acetate buffer (pH 5.6) which would imitate the type of oxidative damage occurring during the release of lysosomes following neutrophil degranulation. The following morning, the reaction was terminated using trichloroacetic acid. The concentration of the end products such as malonic dialdehyde (MDA) and other possible thiobarbituric acid reactive substances (TBARS) was then measured by colorimetric methods [27, 28] using reagents and kits from Cayman Chemical (MC, USA).

### 3.6. Oxidized LDL (LDL-Px)

Activity of serum LDL peroxidase proteins, which include IgG with superoxide dismutase activity, was measured as described previously [29, 30].

### 3.7. Statistics

The results are shown as averages with standard deviation. For the assessment of normally distributed parameters, the Shapiro–Wilk method was used. Student's t-test was then applied both for paired and for unpaired samples. Between-group differences at one time point were evaluated by the Wilcoxon–Mann–Whitney test (continuous variables) and Fisher's exact test (categorical variables). Serum levels of AST and ALT were analyzed using median values and 5% and 95% confidence intervals. AST values are shown as box and whisker plots. Data analysis was performed using Stata (College Station, TX) SE, version 12.1. All statistical tests were two-sided and statistical significance level alpha was set at 0.05 for the analysis.

## 4. Results

### 4.1. Randomization

As can be seen from Table 1, there was a successful randomization of volunteers between the three major groups of the study. No significant differences were seen in gender representation, BMI values, hepatic enzyme levels, serum lipids, and blood pressure parameters in the volunteers enrolled in the study.

### 4.2. Changes in EtOH and AA

Table 2 shows that ingestion of alcohol (0.5 ml/kg or 1.0 ml/kg) resulted in the increase of serum EtOH concentration reflecting the alcohol dose and time of postingestion period. However, at the same dose level changes in the serum EtOH concentration after 1 hour of ingestion were similar in quantitative terms regardless of group identity. In particular, ingestion of alcohol at 0.5 ml/kg gave a similar increase in mean values for EtOH between 455.31 and 511.67 mg/l among the three groups of volunteers. Therefore, none of the interventions seem to affect EtOH absorption from the gastrointestinal tract. As expected, a higher dose of alcohol ingested (1.0 ml/kg) was translated into higher EtOH build-up in the serum (approximately 2-fold higher than at dose of 0.5 ml/kg, Table 1). Interesting changes were seen in the EtOH concentration during the postabsorption period. There was a ~10-fold reduction in the serum EtOH level in all three groups of volunteers at 0.5 ml/kg dose at the end of the observational period. However, all subgroups of volunteers ingesting a higher amount of alcohol (1.0 ml/kg) showed a less significant reduction of serum EtOH concentration which translated at the 5th hour of the observational period into only a 2-fold decline from the starting values. Once again, ingestion of PC regardless of the formulation used had no effect on EtOH clearance dynamics. However, there were significant intergroup differences in serum acetaldehyde (AA) level. First of all, as can be seen from Table 2, regardless of group identity, ingestion of alcohol at 1.0 ml/kg dose was accompanied by an approximately 3 times higher level of AA than was seen with a 0.5 ml/kg ingestion dose. Unlike EtOH serum levels, AA concentration in the placebo control group did not significantly decline especially at the higher (1 ml/kg) ingested dose of alcohol. PC-supplementation did not considerably affect AA levels either. However, volunteers supplemented with lycosome formulation of PC showed a significant reduction in serum AA values especially at the end point of the study. In particular, serum AA in the PC+Lyc group at the 5th hour of the postingestion period (0.5 ml/kg and 1.0 ml/kg) was 58.2% and 25.0% lower than the corresponding values for the placebo group. Overall, unlike EtOH concentration dynamics, a perceptible clearance of AA was seen only in the PC+Lyc group at the end point of the study.

### 4.3. Changes in Total Antioxidant Capacity (TAC)

Table 3 shows changes in serum TAC levels in volunteers. As can be seen below, alcohol intake leads to a statistically significant reduction of serum TAC values in the placebo-treated volunteers at the end of the observational period. In particular, TAC values were reduced by 18.5% and 16.1% in both placebo groups ingesting 0.5 and 1.0 ml/kg of alcohol, respectively. In contrast, supplementation with regular PC prevented such a decline and resulted in a measurable increase of serum TAC values at the 2.5 hours' and 5 hours' time points of the observational period. However, ingestion of lycosome-formulated PC was the most effective in the upregulation of serum TAC values after alcohol intake. There were 38.8% and 29.0% increases in serum TAC in the PC-Lyc group ingesting 0.5 and 1.0 ml/kg of alcohol, respectively, at the 5 hours' time point. A smaller but still measurable increase was seen at the midpoint of the study (2.5 hours).

### 4.4. Changes in MDA and LDL-Px

There was a time-dependent decline in serum MDA level in the placebo-treated volunteers subjected to alcohol intake (Table 4). A higher amount of alcohol ingested (1.0 ml/kg) was accompanied by a more significant decline in serum MDA. Supplementation with regular PC intensified the reduction of serum MDA levels at the end point of the study especially in the group of volunteers ingesting 1.0 ml/kg of alcohol. Nevertheless, the most significant reduction in serum MDA values took place in the participants of the study supplemented with the lycosome formulation of PC. In particular, there was a 62.0% reduction in serum MDA in the volunteers ingesting 0.5 ml/kg of alcohol at the end of the observational period, while the individuals ingesting a higher amount of alcohol (1.0 ml/kg) showed a 78.2% decrease in MDA serum level at the same time point.

A different pattern of changes was seen in serum concentration of oxidized LDL (Table 4). There was a step-wise increase of serum oxidized LDL in placebo-treated volunteers after alcohol ingestion, especially at the alcohol dose of 1.0 ml/kg (a 21.2% over control value at the 5th hour of the observational period). A corresponding increase in the 0.5 ml/kg group was less significant (11.4%). Supplementation of volunteers with regular PC abolished these changes. There was only a 4.4% increase in serum oxidized LDL at the end point of the study in volunteers ingesting 1.0 ml/kg of alcohol. A similar tendency was seen at the lower dose of alcohol ingested (Table 4). Interestingly, lycosome formulation of PC completely reversed the alcohol-induced increase in oxidized LDL levels. There was a similar 13% decline in oxidized LDL at an alcohol intake dose of 0.5 ml/kg and 1.0 ml/kg.

### 4.5. Liver-Specific Markers, Serum Lipids, and hs-CRP

No statistically significant changes were seen in serum AST levels or in bilirubin serum levels. Changes in serum lipids and hs-CRP were below the level of statistical significance accepted in our study.

**Figure 1:**
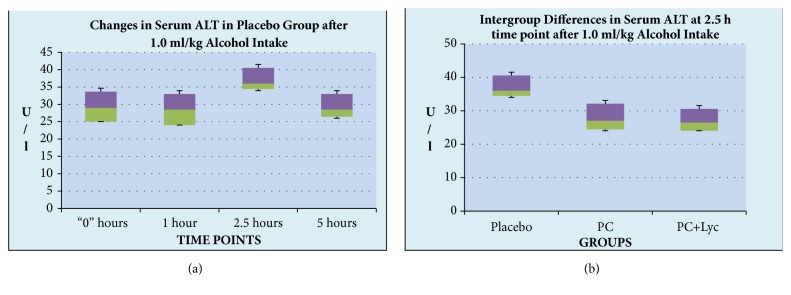
**Time-course ALT changes (a) and intergroup ALT differences (b) after alcohol intake.* Box-and-Whisker Analysis*.** The volunteers were enrolled, screened, and randomized as described in the Material and Methods and given study products as well as alcohol-containing beverages. The parameters were measured at “0” hours and after 1, 2.5, and 5 hours following the intervention.

Similarly, no statistically significant changes were seen in the serum ALT levels with the 0.5 ml/kg alcohol challenge. However, there was a small but measurable increase in serum ALT activity in the volunteers following intake of 1.0 gr/kg of alcohol.  Figure 1(a) shows that after 2.5 hours following intake of alcohol at a dose of 1.0 gr/kg there was an increase in the median ALT values (up to 36.0 IU, 95/5%% CIs: 34.4/40.55) as compared to the zero time point (29.00 IU, 95/5%% CIs:25.00/33.65, P=0.034). Interestingly, such an increase did not take place in the PC- supplemented groups (P>0.05).

## 5. Discussion

The main conclusion from the work presented above is that supplementation of the volunteers with lycopene, a powerful antioxidant, and phosphatidylcholine (PC), a membrane-stabilizing agent, ingested as a highly bioavailable combined lycosome formulation before single intake of a moderate amount of alcohol, ameliorates the increase in serum level of acetaldehyde without affecting circulating levels of ethanol and corrects some abnormalities of biological oxidation caused by alcohol intake.

Moreover, as we have shown above, even single ingestion of a moderate amount of alcohol leads to a state of antioxidant deficiency, as reflected by the decline in TAC of serum and the increase in serum oxidized LDL. Interestingly, these changes developed in a manner reflecting the dose of alcohol as well as time of postabsorption period and could be prevented by two microencapsulated nutraceuticals (PC and lycopene) ingested as a highly bioavailable formulation before alcohol intake. Most importantly, single ingestion of a moderate amount of alcohol under the study conditions was not accompanied by signs of hepatocyte damage. There was only a small and transient increase in AST levels preventable by ingestion of either regular PC or lycosome formulation of PC and lycopene.

First of all, it has to be stated from the beginning that there is a compelling scientific rationale for the use of PC and lycopene in ALD. Both compounds are powerful antioxidants warranting their use in alleviation of the ethanol-induced hepatic oxidative burst which is a keystone characteristic of ALD [14–16]. Moreover, ALD is known to be characterized by limited bioavailability of PC and choline in the liver arising from the ability of ethanol to inhibit hepatic methionine synthase which ultimately results in the suppression of PC biosynthesis in the liver [31–33].

The liver is known to metabolize nearly 90% of ingested ethanol [34]. As we have shown above there is a clear dose-dependency pattern in the dynamics of ethanol clearance. The lower dose of alcohol (0.5 ml/kg) is accompanied by a moderate and transient increase in serum EtOH and AA levels, whereas ingestion of the higher dose of alcohol (1.0 ml/kg) leads to a sustained surge in serum EtOH and AA concentrations. There is a two-step hepatic enzymatic conversion pathway for ethanol [35]. First, the ethanol molecule is converted into acetaldehyde (AA), a highly toxic metabolite mediating alcohol “hangover” symptoms (headache, nausea, and tachycardia), by hepatic cytosolic alcohol dehydrogenase, ADH. Further, AA is transformed into acetic acid, a nontoxic metabolite [35, 36], by mitochondrial aldehyde dehydrogenase type 2 (ALDH-2) highly expressed in liver and to a lesser extent in other tissues (muscles, brain). Although we did not measure activity of ADH and ALDH-2 in the subjects enrolled, the pattern of changes in serum EtOH and AA concentrations seen in the volunteers allows us to make some projections. It is believed [34] that reduction of AA in blood coexisting with unchanged levels of EtOH can be suggestive of more efficient ALDH-2-mediated hepatic enzymatic conversion of AA into acetate. Therefore, it is possible to assume that supplementation with highly bioavailable PC and lycopene increases ALDH-2 activity in the liver and possibly other tissues. Such an assumption becomes more plausible due to the fact that tomato juice constituents increase ALDH-2 activity, a NAD-dependent enzyme, by improving NAD/NADH ratio as well as increasing hepatic pyruvate level [37]. However, according to our results even supplementation of volunteers with PC alone was capable of reducing serum AA values at the end of the observational period (5th hour, 1 ml/kg of alcohol intake). In this sense it has to be mentioned that ALDH-2 is a mitochondrial matrix-associated enzyme; therefore a direct mitochondria-stabilizing effect of PC [38, 39] may contribute to the improved AA turnover seen in volunteers supplemented with PC alone and PC-lycopene formulation before alcohol intake.

On the other hand, it has recently been shown [40] that, besides reducing AA levels, ALDH-2 activation triggers various antioxidant pathways in the early phase of EtOH intoxication and lowers AA-induced reactive oxygen species (ROS) production via activation of PI3K/AKT, SIRT, and CYP2E1 pathways [41] which is consistent with the dose- and time-dependent decline in malonic dialdehyde seen in our study in the placebo-treated treated groups of volunteers. Our results allow us to suggest that the decrease in MAD levels takes place, at least partially, at the expense of depletion of total serum antioxidant capacity simultaneously observed in the placebo groups of the study. The decline in MDA serum level following single alcohol ingestion was a rather unexpected outcome in our study considering multiple references on activation of ROS production in ALD [41, 42]. However, unchanged or decreased levels of hepatic and serum MDA after acute alcohol exposure have been reported before by others [42–44]. In our view the changes in serum MDA and other oxidative stress markers following alcohol intake reflect the dose and multiplicity of alcohol challenge and are likely to follow a biphasic pattern. It seems that the initial activation of ROS production in abstinent individuals after single dose alcohol intake might be well covered by depletion of an endogenous antioxidant pool and activation of ALDH-2 mediated antiradical mechanisms and can be prevented by preingestion of antioxidants. However, prolonged and/or repeated alcohol insults, characterized by sustained AA increase and appearance in the liver of new secondary EtOH metabolites (advanced glycation end-products and others), may activate proinflammatory hepatic pathways and release of ROS from Kupffer and stellate cells leading altogether to increased production of MDA [45, 46]. It is interesting that lycosome formulation of PC and lycopene gave a more significant reduction in serum AA, MDA, and oxidized LDL than PC alone at both doses of alcohol intake (Table 2). This raises a question about possible synergism between PC and lycopene in regulation of hepatic metabolism of ethanol and suggests an improved bioavailability of these nutraceuticals when ingested in the form of lycosomes. First of all, it has to be mentioned that both compounds—PC and lycopene—have distinct antioxidant properties which explains their effect on TAC. PC contains at least one unsaturated fatty acid and choline, which are two antiradical constituents [47], while antioxidant activity of lycopene is mediated by eleven conjugated double bonds in its tetraterpene structure [48]. Therefore, their coingestion as ingredients of lycosome formulation of PC may result in the potentiation of their effects on ethanol conversion and oxidative metabolism.

Our study has obvious limitations. First of all, without isotope analysis, the lycosome-derived PC and lycopene are chemically indistinguishable by means of conventional chemistry from endogenously synthesized analogs circulating in blood. Thus, increased TAC values in volunteers after ingestion of lycosome formulation of PC are the only available but still reasonably informative indication of enhanced bioavailability of PC and lycopene. Secondly, there is a distinct ethnicity pattern in the response to alcohol as well as obvious regional differences in dietary and antioxidant status [49]. Therefore, the relevance of ethnic and nutritional status of volunteers after single intake of alcohol requires vigilant consideration in future studies. Thirdly, as we have shown above, even single ingestion of a moderate amount of alcohol leads to an increase in oxidized LDL level which contradicts epidemiological results regarding the cardioprotective effect of alcohol in moderate alcohol consumers. The significance of this increase in a parameter of cardiovascular health needs to be addressed in future work. And, finally, the pharmacokinetic parameters of PC and lycopene ingested as lycosome microparticles should be directly assessed in prospective studies by isotope/isomer analysis.

## Figures and Tables

**Table 1:** **baseline characteristics (averages±SD).** The volunteers were enrolled, screened, and randomized as described in the Material and Methods. The baseline parameters were measured at day “0” of clinical trial before ingesting the study products.

**VARIABLE**	**GROUPS **		
	**Placebo **	**PC **	**PC+Lyc **
Number of Patients	10	10	10

Males	5	6	5

Females	5	4	5

Age	49.1 ± 4.8	52.3±3.9	53.9±4.2

Light/Moderate Smokers	4	3	2

Body Mass Index	23.5 ± 1.4	22.6±1.7	21.4±1.6

AST in U/L	26.1 ± 6.8	27.4±5.1	29.3±6.3

ALT in U/L	29.5 ± 5.6	25.7±6.4	26.8±4.6

Fasting Glucose in mmol/L	5.9 ± 0.4	5.9±0.3	5.4±0.6

Total Cholesterol in mg/Dl	212.0±11.3	198.5±8.9	215.6±12.3

Triglycerides in mg/Dl	128.0±12.6	115.6±5.7	130.9±7.8

LDL in mg/Dl	120.3 ±8.2	131.4±7.3	133.9±11.7

HDL in mg/Dl	43.3 ± 3.9	46.3±1.7	44.7±2.0

Pulse rate per min	71.8 ± 4.0	69.3±3.5	74.3±4.7

Blood Pressure in mm Hg			

Systolic	120.1±3.9	114.8±5.1	112.0±6.3

Diastolic	77.8±3.2	71.4±2.8	69.9±3.3

**Table 2:** **Serum ethanol (EtOH) and acetaldehyde (AA) values (averages+/- SD).** The volunteers were enrolled, screened, and randomized as described in the Material and Methods and given study products as well as alcohol-containing beverages. The parameters were measured after 1, 2.5, and 5 hours following the intervention.

**Groups **		**0.5 ml/kg **			**1.0 ml/kg **	
	**1 h **	**2.5h **	**5 h **	**1 h **	**2.5 h **	**5 h **
			Et OH mg/l			

**Placebo **	455.31±25.44	314.27±17.34*∗*	45.28±12.19*∗*	1121.87±37.89	1010.39±25.59*∗*	539.21±27.34*∗*

**PC **	487.29±18.37	351.89±21.58*∗*	40.22±14.31*∗*	1235.54±29.14	931.56±17.45*∗*	647.24±24.76*∗*

**PC+Lyc**	511.67±26.72	326.43±19.98*∗*	54.24±17.22*∗*	1175.86±27.76	972.33±21.56*∗*	621.56±21.69*∗*

			A A mg/l			

**Placebo **	1.12±0.14	0.87±0.15*∗*	0.79±0.09*∗*	2.86±0.19	2.99±0.16	2.76±0.21*∗*

**PC **	1.33±0.17	0.94±0.12*∗*	0.83±0.11*∗*	2.88±0.21	2.67±0.19	2.41±0.18*∗*

**PC+Lyc **	0.75±0.12	0.57±0.11*∗*	0.33±0.12*∗*	2.97±0.17	2.34±0.20*∗*	2.07±0.15*∗*

*∗* P<0.05 as compared to “1-hour” time point

**Table 3:** C**hanges in total antioxidant capacity (TAC) of serum.** The volunteers were enrolled, screened, and randomized as described in the Material and Methods and given study products as well as alcohol-containing beverages. The parameters were measured at “0” hours and after 1, 2.5, and 5 hours following the intervention.

**TAC (mM TE/L) Averages±SD**				
	**Time Points:**			
**Groups**	“0” Hour	1 h	2.5 h	5 h

Alcohol Intake 0. 5 ml/kg				

Placebo	1.78±0.11	1.80±0.13	1.61±0.15	1.45±0.15*∗*

PC	1.69±0.09	1.79±0.13	1.90±0.08*∗*	2.15±0.12*∗*

PC+Lyc	1.70±0.10	1.93±0.12*∗*	2.12±0.14*∗*	2.36±0.16*∗*

Alcohol Intake 1.0 ml/kg				

Placebo	1.67±0.10	1.60±0.13	1.53±0.16	1.40±0.12

PC	1.74±0.13	1.70±0.09	1.95±0.11*∗*	2.03±0.18*∗*

PC+Lyc	1.79±0.12	1.82±0.14*∗*	2.20±0.15*∗*	2.31±0.20*∗*

*∗* P<0.05 as compared to “0-hour” time point

**Table 4:** **Changes in serum malonic dialdehyde (MDA) and oxidized LDL (LDL-Px) levels (averages +/-SD).** The volunteers were enrolled, screened, and randomized as described in the “Material and Methods” Section and given study products as well as alcohol-containing beverages. The parameters were measured at “0” hours and after 1, 2.5, and 5 hours following the intervention.

	**MDA **µ**M**				**LDL-Px (E-460)**				
	**Time Points:**					**Time Points:**			
**Groups**	“0” Hour	1 h	2.5 h	5 h	**Groups**	“0” Hour	1 h	2.5 h	5 h

**Alcohol Intake 0.5 ml/kg**									

**Placebo **	88.1±3.1	77.9±2.7*∗*	69.9±2.6*∗*	49.9±1.7*∗*	**Placebo**	218.9±3.6	225.6±1.7*∗*	237.4±2.8*∗*	243.4±3.6*∗*

**PC **	90.4±3.3	71.3±1.9*∗*	60.5±3.2*∗*	40.3±2.5*∗*	**PC**	223.4±2.3	227.6±2.1*∗*	230.1±2.2*∗*	228.4±2.7*∗*

**PC+Lyc **	87.3±2.9	66.0±1.6*∗*	48.1±2.0*∗*	25.1±2.3*∗*	**PC+Lyc**	225.6±2.7	218.0±3.4*∗*	210.4±3.0*∗*	195.4±2.9*∗*

**Alcohol Intake 1.0 ml/kg**									

**Placebo **	93.4±1.8	65.4±2.6*∗*	57.3±2.5*∗*	35.4±2.0*∗*	**Placebo**	216.7±2.7	235.1±1.6*∗*	245.6±1.6*∗*	261.9±4.5*∗*

**PC **	91.6±2.3	57.4±1.9*∗*	54.2±2.6*∗*	30.3±3.1*∗*	**PC**	221.9±3.1	225.6±2.0	227.6±2.1*∗*	231.7±3.7*∗*

**PC+Lyc **	89.4±1.2	53.1±2.1*∗*	42.3±1.4*∗*	19.3±2.6*∗*	**PC+Lyc**	220.0±2.6	210.7±1.9*∗*	207.0±3.6*∗*	190.4±2.9*∗*

## Data Availability

The supporting results will be displayed on publicly available website Lycotec.com. Moreover, the data that support the findings of this study are available from the corresponding author, Yuriy K. Bashmakov, upon reasonable request.

## References

[1] Llerena S., Arias-Loste M. T., Puente A., Cabezas J., Crespo J., Fábrega E. (2015). Binge drinking: burden of liver disease and beyond. *World Journal of Hepatology*.

[2] Chick J. (2011). The WHO global strategy to reduce the harmful use of alcohol. *Alcohol and Alcoholism*.

[3] Zaridze D., Brennan P., Boreham J. (2009). Alcohol and cause-specific mortality in Russia: a retrospective case-control study of 48 557 adult deaths. *The Lancet*.

[4] Levitt M. D., Levitt D. G., Furne J., DeMaster E. G. (1994). Can the liver account for first-pass metabolism of ethanol in the rat?. *American Journal of Physiology*.

[5] Poole L. G., Dolin C. E., Arteel G. E. (2017). Organ-organ crosstalk and alcoholic liver disease. *Biomolecules*.

[6] Duseja A., Singh S. P. (2017). Toward a better definition of acute-on-chronic liver failure. *Journal of Clinical and Experimental Hepatology*.

[7] Rana R., Wang S. L., Li J., Xia L., Song M. Y., Yang C. Q. (2017). A prognostic evaluation and management of alcoholic hepatitis. *Minerva Medica*.

[8] Stickel F., Datz C., Hampe J., Bataller R. (2017). Pathophysiology and management of alcoholic liver disease: update 2016. *Gut and Liver*.

[9] Farooq M. O., Bataller R. (2016). Pathogenesis and management of alcoholic liver disease. *Digestive Diseases*.

[10] Ramadori P., Cubero F. J., Liedtke C., Trautwein C., Nevzorova Y. A. (2017). Alcohol and hepatocellular carcinoma: adding fuel to the flame. *Cancers*.

[11] Xu M., Zhou Z., Parker R., Gao B. (2017). Targeting inflammation for the treatment of alcoholic liver disease. *Pharmacology & Therapeutics*.

[12] Pinzani M., Luong T. V. (2018). Pathogenesis of biliary fibrosis. *Biochimica et Biophysica Acta (BBA)—Molecular Basis of Disease*.

[13] Singh S., Osna N. A., Kharbanda K. K. (2017). Treatment options for alcoholic and non-alcoholic fatty liver disease: a review. *World Journal of Gastroenterology*.

[14] Ghorbani Z., Hajizadeh M., Hekmatdoost A. (2016). Dietary supplementation in patients with alcoholic liver disease: a review on current evidence. *Hepatobiliary & Pancreatic Diseases International*.

[15] Ma Z., Zhang Y., Li Q., Xu M., Bai J., Wu S. (2017). Resveratrol improves alcoholic fatty liver disease by downregulating HIF-1*α* expression and mitochondrial ROS production. *PLoS ONE*.

[16] Nagy L. E., Ding W.-X., Cresci G., Saikia P., Shah V. H. (2016). Linking pathogenic mechanisms of alcoholic liver disease with clinical phenotypes. *Gastroenterology*.

[17] Petyaev I. M. (2016). Lycosome technology: advances and perspectives. *American Journal of Food Science and Nutrition*.

[18] Petyaev I. Carotenoid particles and uses thereof.

[19] Petyaev I. M. (2015). Improvement of hepatic bioavailability as a new step for the future of statin. *Archives of Medical Science*.

[20] Petyaev I. M., Dovgalevsky P. Y., Chalyk N. E., Klochkov V., Kyle N. H. (2014). Reduction in blood pressure and serum lipids by lycosome formulation of dark chocolate and lycopene in prehypertension. *Food Science & Nutrition*.

[21] Bashmakov Y. K., Assaad-Khalil S. H., Abou Seif M. (2014). Resveratrol promotes foot ulcer size reduction in type 2 diabetes patients. *ISRN Endocrinology*.

[22] Petyaev I. M., Dovgalevsky P. Y., Klochkov V. A., Chalyk N. E., Kyle N. (2012). Whey protein lycosome formulation improves vascular functions and plasma lipids with reduction of markers of inflammation and oxidative stress in prehypertension. *The Scientific World Journal*.

[23] Ivan M., Petyaev P, Bandaletova T., Natalia E., Klochkov V., Nigel H. (2016). Lycosome formulation of dark chocolate increases absorption cocoa catechins and augments their anti-inflammatory and antioxidant properties. *American Journal of Food Science and Nutrition*.

[24] Petyaev I. M., Bashmakov Y. K. (2017). Dark chocolate: opportunity for an alliance between medical science and the food industry?. *Frontiers in Nutrition*.

[25] Pontes H., De Pinho P. G., Casal S. (2009). GC determination of acetone, acetaldehyde, ethanol, and methanol in biological matrices and cell culture. *Journal of Chromatographic Science (JCS)*.

[26] Kozutsumi D., Arita M., Kawashima A., Adachi M., Takami M. (2002). An improved method for acetaldehyde determination in blood by high-performance liquid chromatography and solid-phase extraction. *Journal of Chromatographic Science (JCS)*.

[27] Ghani M. A., Barril C., Bedgood D. R., Prenzler P. D. (2017). Measurement of antioxidant activity with the thiobarbituric acid reactive substances assay. *Food Chemistry*.

[28] Moore K., Roberts L. J. (1998). Measurement of lipid peroxidation. *Free Radical Research*.

[29] Petyaev I., Mitchinson M. M. J., Hunt J. V., Coussons P. J. (1998). Superoxide dismutase activity of antibodies purified from the human arteries andatherosclerotic lesions. *Biochemical Society Transactions*.

[30] Petyaev I. M., Coussons P. J. (1999). Superoxide dismutase: recent advances and clinical applications. *Superoxide dismutase activity of antibodies purified from human atherosclerotic lesions*.

[31] Barak A. J., Beckenhauer H. C., Tuma D. J. (2002). Methionine synthase: a possible prime site of the ethanolic lesion in liver. *Alcohol*.

[32] Obeid R. (2013). The metabolic burden of methyl donor deficiency with focus on the betaine homocysteine methyltransferase pathway. *Nutrients*.

[33] Wang F., Zhang Y., Zhou Y. (2016). Effects of beverages on alcohol metabolism: potential health benefits and harmful impacts. *International Journal of Molecular Sciences*.

[34] Cederbaum A. I. (2012). Alcohol metabolism. *Clinics in Liver Disease*.

[35] Dinis-Oliveira R. J. (2016). Oxidative and non-oxidative metabolomics of ethanol. *Current Drug Metabolism*.

[36] Ushida Y., Oshima S., Aizawa K. (2014). Aqueous components of tomato accelerate alcohol metabolism by increasing pyruvate level. *Journal of Food and Nutrition Sciences*.

[37] Stickel F., Moreno C., Hampe J., Morgan M. Y. (2017). The genetics of alcohol dependence and alcohol-related liver disease. *Journal of Hepatology*.

[38] Schuler M.-H., Di Bartolomeo F., Mårtensson C. U., Daum G., Becker T. (2016). Phosphatidylcholine affects inner membrane protein translocases of mitochondria. *The Journal of Biological Chemistry*.

[39] Xue L., Xu F., Meng L. (2012). Acetylation-dependent regulation of mitochondrial ALDH2 activation by SIRT3 mediates acute ethanol-induced eNOS activation. *FEBS Letters*.

[40] Steiner J. L., Lang C. H. (2017). Etiology of alcoholic cardiomyopathy: mitochondria, oxidative stress and apoptosis. *The International Journal of Biochemistry & Cell Biology*.

[41] Yuan R., Tao X., Liang S. (2018). Protective effect of acidic polysaccharide from Schisandra chinensis on acute ethanol-induced liver injury through reducing CYP2E1-dependent oxidative stress. *Biomedicine & Pharmacotherapy*.

[42] Antonenkov V. D., Pirozhkov S. V., Popova S. V., Panchenko L. F. (1989). Effect of chronic ethanol treatment on lipid peroxidation in rat liver homogenate and subcellular fractions. *International Journal of Biochemistry*.

[43] Antonenkov V. D., Pirozhkov S. V., Popova S. V., Panchenko L. F. (1990). The effect of ethanol and the catalase inhibitor 3-amino-1, 2, 4-triazole on lipid peroxidation in rat liver homogenate and subcellular fractions. *Voprosu Medicinskoi Khimii*.

[44] Khokha A. M., Kashko M. F., Antsulevich S. N., Doroshkevich N. A., Voronov P. P. (1993). Effect of acute alcohol intoxication on lipid peroxidation in testis and adrenal glands of rats. *Voprosy Pitaniia*.

[45] Takeuchi M., Takino J.-I., Sakasai-Sakai A., Takata T., Tsutsumi M. (2017). Toxic AGE (TAGE) theory for the pathophysiology of the onset/progression of NAFLD and ALD. *Nutrients*.

[46] Baldi E., Burra P., Plebani M., Salvagnini M. (1993). Serum malondialdehyde and mitochondrial aspartate aminotransferase activity as markers of chronic alcohol intake and alcoholic liver disease. *Italian Journal of Gastroenterology*.

[47] Abbasi I. H. R., Abbasi F., Soomro R. N. (2017). Considering choline as methionine precursor, lipoproteins transporter, hepatic promoter and antioxidant agent in dairy cows. *AMB Express*.

[48] Petyaev I. M. (2016). Lycopene deficiency in ageing and cardiovascular disease. *Oxidative Medicine and Cellular Longevity*.

[49] Roerecke M., Nanau R., Rehm J., Neuman M. (2016). Ethnicity matters: a systematic review and meta-analysis of the non-linear relationship between alcohol consumption and prevalence and incidence of hepatic steatosis. *EBioMedicine*.

